# The effectiveness of intrauterine antibiotic infusion versus oral antibiotic therapy in the treatment of chronic endometritis in patients during IVF (in vitro fertilization) procedures

**DOI:** 10.1186/s12905-022-02128-8

**Published:** 2022-12-17

**Authors:** Mihai Luncan, Anca Huniadi, Erika Bimbo-Szuhai, Mihai Botea, Ioana Zaha, Liana Stefan, Corina Beiusanu, Dana Romanescu, Annamaria Pallag, Alin Bodog, Laurean Ovidiu Pop, Mircea Ioan Șandor

**Affiliations:** 1 Faculty of Medicine and Pharmacy, University of Oradea, 1St December Square 10, 410073 Oradea, Romania; 2 Pelican Clinical Hospital, Corneliu Coposu Street, 2, 41045Ö, Oradea, Romania; 3 Calla-Infertility Diagnostic and Treatment Center, Constantin A. Rosetti Street, 410103 Oradea, Romania; 4 Department of Pharmacy, Faculty of Medicine and Pharmacy, University of Oradea, 29 Nicolae Jiga Street, 410028 Oradea, Romania

**Keywords:** Chronic endometritis, Hysteroscopy, Immunohistochemistry for CD 138, Intrauterine antibiotic infusion, Oral antibiotic therapy

## Abstract

**Background:**

Chronic Endometritis (CE) is a subtle pathology, likely infectious in most cases, with a negative impact on the female fertility, but often overlooked even among fertility specialists. The purpose of the study is to demonstrate the predominant infectious nature of CE and to find the best therapeutic option by comparing the results of oral antibiotic therapy versus intrauterine antibiotic infusion in patients with CE undergoing IVF procedures. The objective was to compare the cure rate of CE—defined as the percentage of patients without CE at the test of cure, between the two groups and, the hysteroscopic aspect with the positive CD 138 staining.

**Methods:**

This was a prospective, case—control study that took place in a single university fertility clinic, in Oradea, Romania and included 57 patients with CE divided into 2 groups: orally administered antibiotics group who received a combination of antibiotics compared to intrauterine infusion group who received intrauterine infusion of antibiotic. Chronic Endometritis was diagnosed through hysteroscopy and immunohistochemistry for CD 138. Patients in both groups were tested for CE twice to evaluate the cure rate after oral combination antibiotic therapy versus intrauterine infusion of antibiotic.

**Results:**

Out of 115 patients with endometrial biopsies 57 tested positive for CE, with a 49.6% chronic endometritis prevalence. Among the group that was administered oral antibiotics, 11 patients (45.83%) experienced CE resolution after triple antibiotic therapy. Of the intrauterine infusion group, 25 patients (89.29%) presented negative results (p 0.0020). The normal hysteroscopic aspect had a similar prevalence in the patients with immunohistochemical positive and negative CD 138.

**Conclusions:**

Our study demonstrated the effectiveness and superiority of intrauterine antibiotic infusion over the use of oral combination antibiotic therapy for CE cure.

*Trial Registration*: ISRCTN17542620/14.09.2022.

## Introduction

Chronic endometritis (CE) is a subtle pathology, probably infectious in most cases, characterized by an inflammatory state of the endometrium caused by various pathogens: common gram-positive (Streptococcus, Staphylococcus), gram-negative (Escherichia Coli, Klebsiella pneumoniae, Neisseria gonorrhoeae), intracellular (Mycoplasma, Ureaplasma, Chlamydiae) or anaerobic (Bifidobacteria, Prevotella) [1]. Chronic endometritis usually is asymptomatic or has inconsistent symptoms such as abnormal bleeding, chronic pelvic pain, dyspareunia or vaginal discharge over a long period of time with the tendency of persistence and slow progression, finally compromising the reproductive potential of patients. Because of these reasons chronic endometritis is rarely taken under consideration even by fertility specialists.

Chronic endometritis is also a diagnostics challenge [2]. Hysteroscopy is a useful tool in diagnosing CE, as there are classic images for CE, but its overall accuracy is operator dependent. Histology also has some limitations and interobserver variability especially because, to date there are no clear recommendations of the criteria for diagnosis of chronic endometritis. The identification of a pathogen that can cause chronic endometritis in endometrial culture is hampered by the exaggerated growth of conditional pathogenic microorganisms. Even the histological identification of plasmacyte by hematoxylin–eosin coloration has its limitations: technically insufficient coloration, incorrect conservation of probes, and stromal proliferative cells with excessive mitosis that can mask plasmacytes and lead to a false negative result. On the other hand false-positive results can occur by confusing plasmacytes with mononuclear cells or stromal plasmacytoid cells [3, 4].

The markers used in the diagnosis of endometritis are MUM1 and Syndecan-1 (or CD 138) [5]. In our study we used CD 138. Syndecan-1 (or CD 138) is a transmembrane heparin proteoglycan on the plasmacyte surface and is considered the immunohistochemical marker used in the diagnosis of chronic endometritis [6]. In comparison with hysteroscopy alone, the immunohistochemical coloration for CD 138 offers a higher specificity and sensitivity with minimal variability between different observers [7, 8]. Even though the diagnosis is histological, hysteroscopy plays a role in determining the specific site of biopsy in chronic endometritis [9]. All being said even the immunohistochemical coloration for CD 138 has its limitations. First of all, the interference of endometrial epithelial cells that express CD 138 can be marked by monoclonal antibodies that are specific for CD 138 [10]. Secondly, the accuracy of immunohistochemical diagnosis can be influenced by the moment of the endometrial biopsy in the menstrual cycle and the quantity of endometrial tissue that is retrieved. Biopsies retrieved in the proliferative phase of the menstrual cycle have bigger chances to detect plasmacytes in comparison with the biopsies taken in the secretory phase of the menstrual cycle [11]. Because of the petechiae characteristics of plasmacyte congestion, these can be overlooked in a small sample [12]. There are variations among diagnostic criteria between different in vitro fertilization centers when it comes to chronic endometritis: in some centers the specimens that present with at least 5 positive CD 138 cells from 1 to 3 of section levels of microtomy are considered positive, but in many centers only 1 CD 138 positive cell in a high power field (HPF) microscope is considered a criteria for a positive diagnosis [4, 12–14].

Most likely due to these differences in definition and positive diagnosis, the prevalence of this chronic illness in women that struggle with infertility is variable: from 0.2 to 46% [6, 15–17]. In studies that were evaluating patients with repeated failure of implantation the percentage was between 14 and 60% [18–20].

At present, there is also a lot of controversies regarding the management of CE: whether to treat or not to treat and how to treat. Being an infectious disease, the treatment for chronic endometritis is based on the administration of antibiotics that are effective against the bacteria that is causing this chronic problem. After the administration of antibiotics we expect an improvement of the inflammatory changes and endometrial receptivity [20–24]. The immune response against bacterial invasion of the uterine cavity during chronic endometritis rarely progresses to a systemic inflammatory response [21], therefore the necessity of systemic antibiotic therapy could be considered but may not be crucial. The local administration of antibiotics (uterine infusion) is described in case reports [22], as well as a recent clinical study [23].

Moreover, a new theory about an impaired inflammatory state of the endometrium relying on multiple pathogenic mechanisms, not only infectious but also hormonal and autoimmune could be involved. This would make things even more complicated about this already controversial pathology [25].

In this prospective case control study we aimed find the prevalence of CE in infertile patients and to demonstrate the infectious nature of CE and to evaluate the effectiveness of oral antibiotic therapy compared with local antibiotic therapy for CE cure.

## Material and method

### Study design

This was a prospective, case–control study, conducted at a single university level fertility center in Oradea, Bihor country, Romania during the period of time between 01.01.2021–31.12.2021. This study was conducted under the principles of the Helsinki declaration and has been approved by the ethics committee of Calla Clinic, Oradea, Romania (463/05.02.2020). All patients included have signed an informed consent for their participation in the study. Information given to patients was about side effects of the procedures.

### Participants

We included patients that underwent an IVF procedure in our clinic and compared women diagnosed with CE who received oral antibiotic with women diagnosed with CE who received local antibiotic therapy (infusions) referred to our clinic from 01 January 2021 to 31 December 2021. The patients had the opportunity to choose whether they follow oral or local antibiotic therapy. All patients underwent a second biopsy to control the efficiency of the treatment. We included only women with hysteroscopy diagnosed CE by immunohistochemistry for CD 138. The diagnosis was based on the demonstration > 1 plasma cells per 10 high power fields according to many published studies [5, 26, 27].

Inclusion criteria were: patients that underwent an IVF procedure in our clinic, having various IVF indications that obtained at least two day 5–6 blastocysts and agreed with a hysteroscopic examination.

Exclusion criteria were: acute endometritis or other acute pelvic inflammatory disease, placental remanence, steroid and antibiotic treatment within 3 months prior to diagnosis, endometrial cancer or atypical hyperplasia and refusal of procedure (hysteroscopy) or treatment for chronic endometritis.

The diagnosis of chronic endometritis is based on a hysteroscopy that is done in the beginning of the menstrual cycle (days 9–11). The guided biopsy during hysteroscopy was then sent for pathological and immunohistochemical examination. In this study the positive diagnosis of chronic endometritis is based on the presence of at least one CD 138 positive plasmacyte per 10 high power fields [2, 10, 15, 16].

#### Data collection

The data of the patients was gathered in our clinic by two authors. The register included: age of the patient, cause of infertility, previous pregnancies, hysteroscopic findings, immunohistochemical diagnostic, the type of treatment that had been administered the negative immunohistochemical test after which treatment.

#### Hysteroscopy and endometrial sampling

Hysteroscopy was done under intravenous general anesthesia, after the disinfection of the perineum with a solution of chlorhexidine and the external cervical ostium with iodine solution after a vaginoscopy to minimize contamination risk. Visualization of the uterine mucosa was done by crossing the cervical canal with a compact hysteroscope CAMPO TROPHYSCOPE® (Karl Storz SE & Co. KG, Germany), with a HOPKINS® 30° telescope, size 2.9 mm, length 24 cm, with an irrigation connector and an operation sheath with continuous flux 26.152 DA and 26.152 DB. Entering the uterine cavity we look for two tubal ostium and a panoramic image of the uterus. With biopsy forceps we draw a mucosal probe from the macroscopical modified region (polyposis endometrium, oedema or hyperemia of endometrial mucosa). If there are no visible endometrial alterations we extract a biopsy with a catheter (Gynetics Medical Products Endometrial Curette) that is crossing under visual control of the cervical canal without any contact with the vaginal wall. The endometrial probes were put in a saline solution (2 mL) and sent to the pathology laboratory for examination.

### Histopathological examination

Evaluation of endometrial biopsies was performed from formalin-fixed paraffin-embedded tissue. Samples were sectioned using a Leica RM 2125 RT microtome and processed using the standard hematoxylin and eosin stain (HE). Tissue analysis was performed using a standardized Leica system for image acquisition and analysis—a DM 3000 LED microscope equipped with K3 camera.

Tissue specimens were fixed in 10% buffered formalin (pH 7.4) for up to 72 h and paraffin-embedded according to the standard procedures. A 4 μm representative section from each paraffin-embedded tissue was performed according to a standard automated immunohistochemical procedure (Autostainer Link 48 DAKO; Agilent, Santa Clara, CA 95.051; United States) [24]. For each case anti-CD138 (cone MI15-DAKO) mouse monoclonal antibody, immunohistochemical profile was determined. Negative controls were processed by the same methodology but omitting the primary antibody. Positive controls consisted of normal tonsil positive for CD138. At least 50 high power fields were examined per specimen and the biopsies were graded as negative for chronic endometritis if there was < 1 plasma cells identified per 10 high power fields and positive when there was > 1 plasma cells identified per 10 high power fields [28].

Acute endometritis typically represents ascending infection from lower genital tract. The most common causes are Chlamydia trachomatis, Neisseria gonorrhoeae, Trichomonas vaginalis and Mycoplasma species.

The differential diagnosis can be done by tissue examination. The acute endometritis reveals the presence of neutrophils infiltrating and destroying endometrial epithelium not plasma cells like in chronic endometritis. Sometimes the neutrophils are organized in microabscess. The different inflammatory cells do the diagnostic easily.

### Treatment of chronic endometritis

The treatment was decided by the patient after a thorough presentation of the method of administration, duration of treatment and side-effects to each patient.

*a. Oral antibiotic treatment—*to have a high range of microorganisms that can cause chronic endometritis covered by the antibiotic we use a combination of 3 antibiotics over a 14 days period of time: ciprofloxacin 500 mg 2 times a day, doxycycline 100 mg 2 times a day and metronidazole 500 mg de 2 times a day [25, 29].

*b. Intrauterine antibiotic infusions—*our protocol for intrauterine infusions includes the administration of 3 ml ciprofloxacin 200 mg/100 ml concentration every 3 days with 10 infusions in total across a 30 day period of time for each patient. Infusions were done using Labotect Embryo Transfer Catheter 23 cm (Labotect Labor-Technik-Gottingen GMBH, Rusdorf, Germany).

### Assessment of CE cure

In the follicular phase of the menstrual cycle following antibiotic therapy all women underwent a repeat endometrial biopsy with CD 138 immunohistochemical examination.

### Statistic analyses

For the storage of information from the study and for the statistical analysis we used a medical statistics program MedCalc® 12.5.0.0 (MedCalc® Software, Mariakerke, Belgium). Statistical results will be represented by the probability of null (p), a p value under 0.05 underlines a significant difference between the studied groups. Every continuous variable will be checked for the value distribution using the Kolmogorov–Smirnov test. Continuous variables with normal distribution will be represented by average and standard deviation in brackets, and the asymmetrical distribution by median and interquartile range in brackets. Also depending on the variable character parametric tests will be used (Student test for independent lots) or non-parametric (Mann–Whitney test). Comparison between categorical variables were tested using contingency tables and the chi-square test or Fisher`s test when necessary.

### Study endpoints

The primary endpoint consisted in obtaining the prevalence of CE in a population that undergoes an IVF procedure. The secondary endpoint was to compare the rate of CE resolution between the two groups. The tertiary endpoint was to compare the hysteroscopic normal appearance with the presence of chronic endometritis.

## Results

**Fig. 1:**
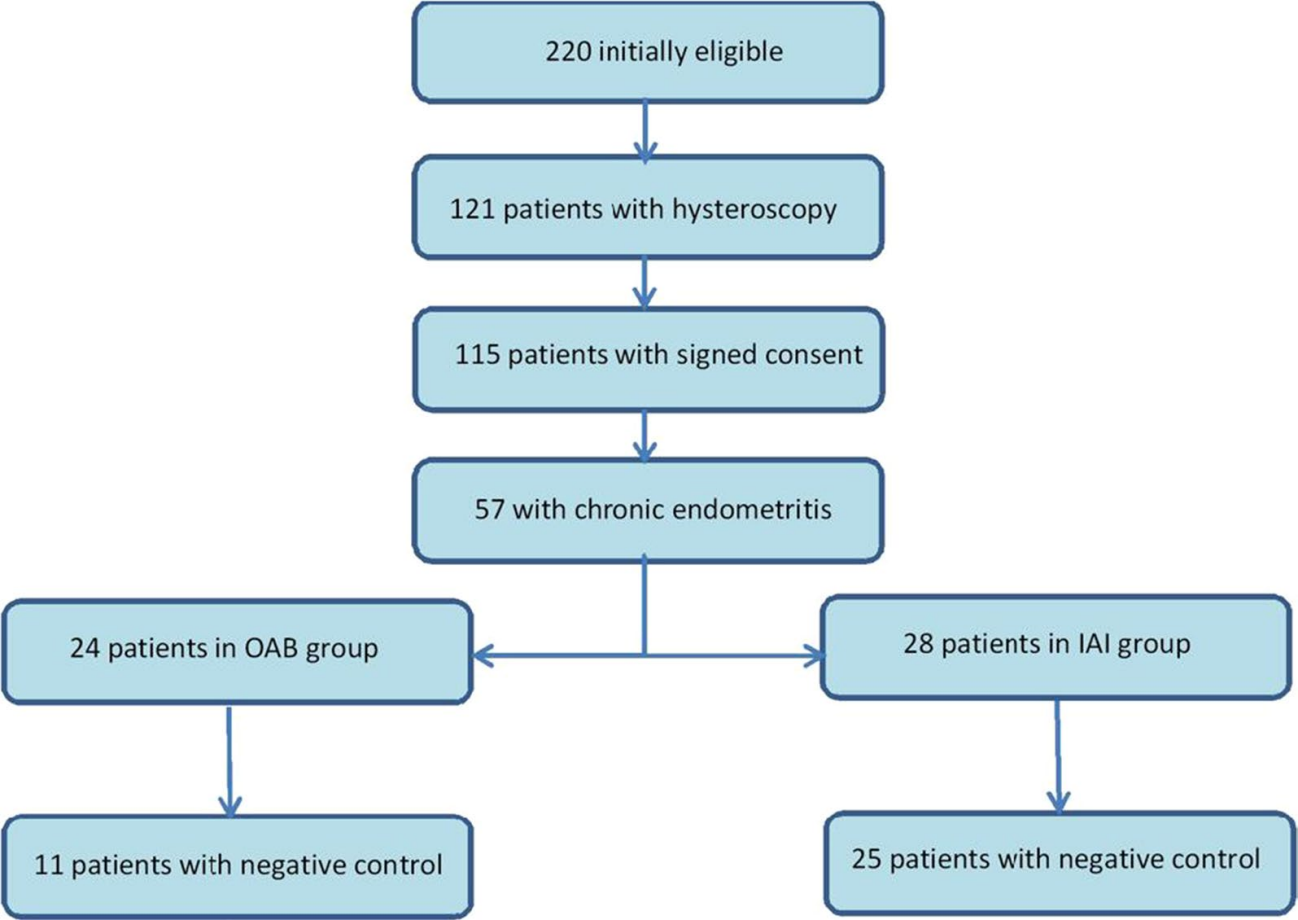
Algorithm of patient selection in the study groups

From the total of 232 patients that presented in the given period of time, 220 remained eligible for treatment, from which only 115 accepted to do the hysteroscopy and the control biopsy. Using the diagnosis criteria 57 cases of chronic endometritis were identified. After they have chosen the treatment 27 patients were included in the oral antibiotic therapy group (OAB) and 30 patients were included in the intrauterine antibiotic infusion group (IAI). Because some patients needed both treatment options to become negative and made part of both groups (the inefficiency of one treatment and changing to the other treatment) 5 more patients were excluded (3 from the OAB group and 2 from the IAI group). The algorithm of the patient selection is shown in Fig. 1.

The demographic characteristics of the study population that underwent an IVF procedure are displayed in Table 1. The age, the provenance, the obstetrical history, the normal hysteroscopic aspect were not significantly different between groups. Regarding the indication for the IVF procedures, diminished ovarian reserve was encountered more frequently in patients without chronic endometritis, but we consider this an incidental aspect.

**Table 1:** Demographic characteristics of the study population—patients with positive and negative diagnosis for chronic endometritis

Variable	With chronic endometritis (n = 57)	Without chronic endometritis (n = 58)	p (statistical significance)
Age (years)—average (± SD)	35.08 (± 4.6)	35.84 (± 5.0)	0.4010
Provenance—U (percentage—%)	38 (66.67%)	36 (62.07%)	0.7490
Previous abortion—Yes (percentage—%)	12 (21.05%)	15 (25.86%)	0.6977
Ectopic pregnancy—Yes (percentage—%)	4 (7.01%)	1 (1.72%)	0.3501
Birth—Yes (percentage—%)	3 (5.26%)	2 (3.45%)	0.9841
Hysteroscopic aspects—normal (percentage—%)	35 (61.40%)	34 (58.62%)	0.9091
IVF indication (%)			
Low AMH	3 (5.26%)	14 (24.14%)	0.0096
PCOS	0 (0%)	2 (3.45%)	0.4833
Tubal	11 (11.29%)	7 (12.07%)	0.4179
Advanced maternal			
Age	13 (22.80%)	7 (12.07%)	0.2030
FF	14 (24.56%)	15 (25.86%)	0.9568
MF	16 (28.07%)	15 (25.86%)	0.9548
Unknown	12 (21.05%)	12 (20.69%)	0.8559

*SD* = standard deviation; *U* = urban; *R* = rural; *IVF* = in vitro fertilization; Anti mullerian hormone (AMH) = diminished ovarian reserve; *PCOS* = polycystic ovarian syndrome; *FF* = feminine factor; *MF* = masculine factor

Chronic endometritis prevalence according to the diagnosis criteria is 49.6%. After the diagnosis patients were divided in the oral antibiotic therapy group (OAB) and the intrauterine antibiotic infusion group (IAI). The division of patients according to the treatment option gave us the following results in clinical and demographic characteristics (Table 2):

**Table 2:** Demographic and clinical characteristics for the 2 groups of patients according to the treatment option for chronic endometritis

Characteristics	Treatment OAB (n = 24)	Treatment IAI (n = 28)	p (statistical semnification)
Age (years)—average (± DS)	34.92 (± 4.2)	35.14 (± 5.0)	0.8629
Provenience—U (percentage—%)	14 (58.33%)	19 (67.86%)	0.6729
After treatment—negative (%)	11 (45.83%)	25 (89.29%)	**0.0020**
IVF results (%)			
Ongoing pregnancy	5 (20.83%)	13 (46.43%)	0.1007
Abortion	3 (12.50%)	2 (7.14%)	0.8560
Birth	1 (4.17%)	0 (0.00%)	0.9379

*OAB* = oral antibiotic; *IAI* = Intrauterine antibiotic infusion; *SD* = standard deviation; *U* = urban; *R* = rural; *IVF* = in vitro fertilization;

Among oral antibiotic group, 11 patients (45.83%) experienced CE resolution after the triple antibiotic therapy. Among intrauterine infusion group, 25 patients (89.29%) presented negative controls (p 0.0020). The efficiency of the treatment in producing negative CD 138 testing was significantly higher for the patients treated by the intrauterine antibiotic infusion: RR = 1.9481 (IC95%: 1.2–3.06), p = 0.0039 with NNT = 2.301 (IC95%: 1.52–4.71).

**Fig. 2:**
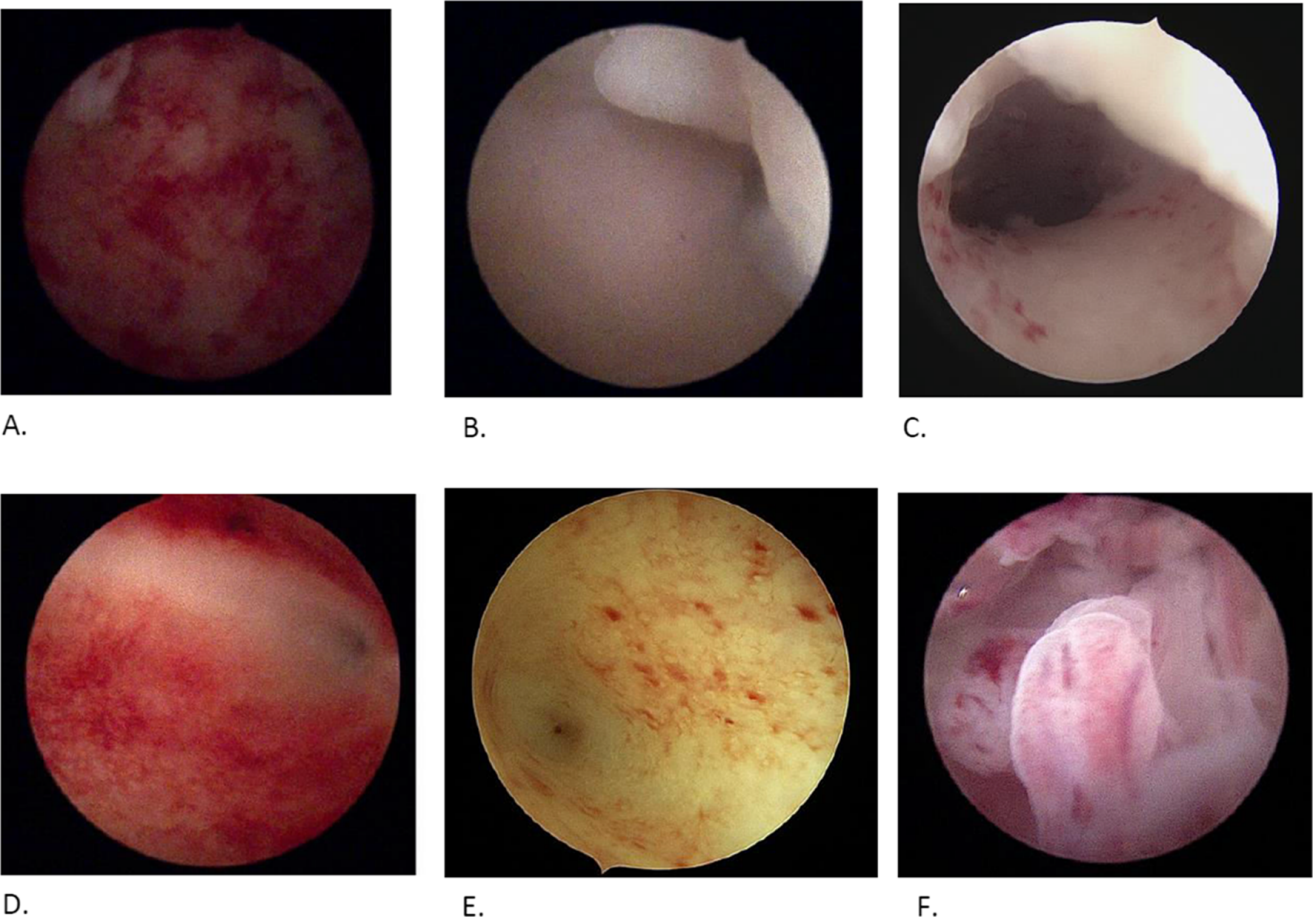
Hysteroscopy findings in chronic endometritis. **A**. Generalized hyperemia. **B**. Pale mucosa with polyp. **C**. Pale mucosa with hemorrhagic spots. **D**. Strawberry aspect. **E**. Focal hyperemia. **F**. Micro-polyps

The hysteroscopic aspects of chronic endometritis are considered to be the classical strawberry pattern but also hemorrhagic spots, focal and generalized hyperemia, pale and edematous mucosa and the presence of micro-polyps as shown in the figure bellow (Fig. 2).

**Fig. 3:**
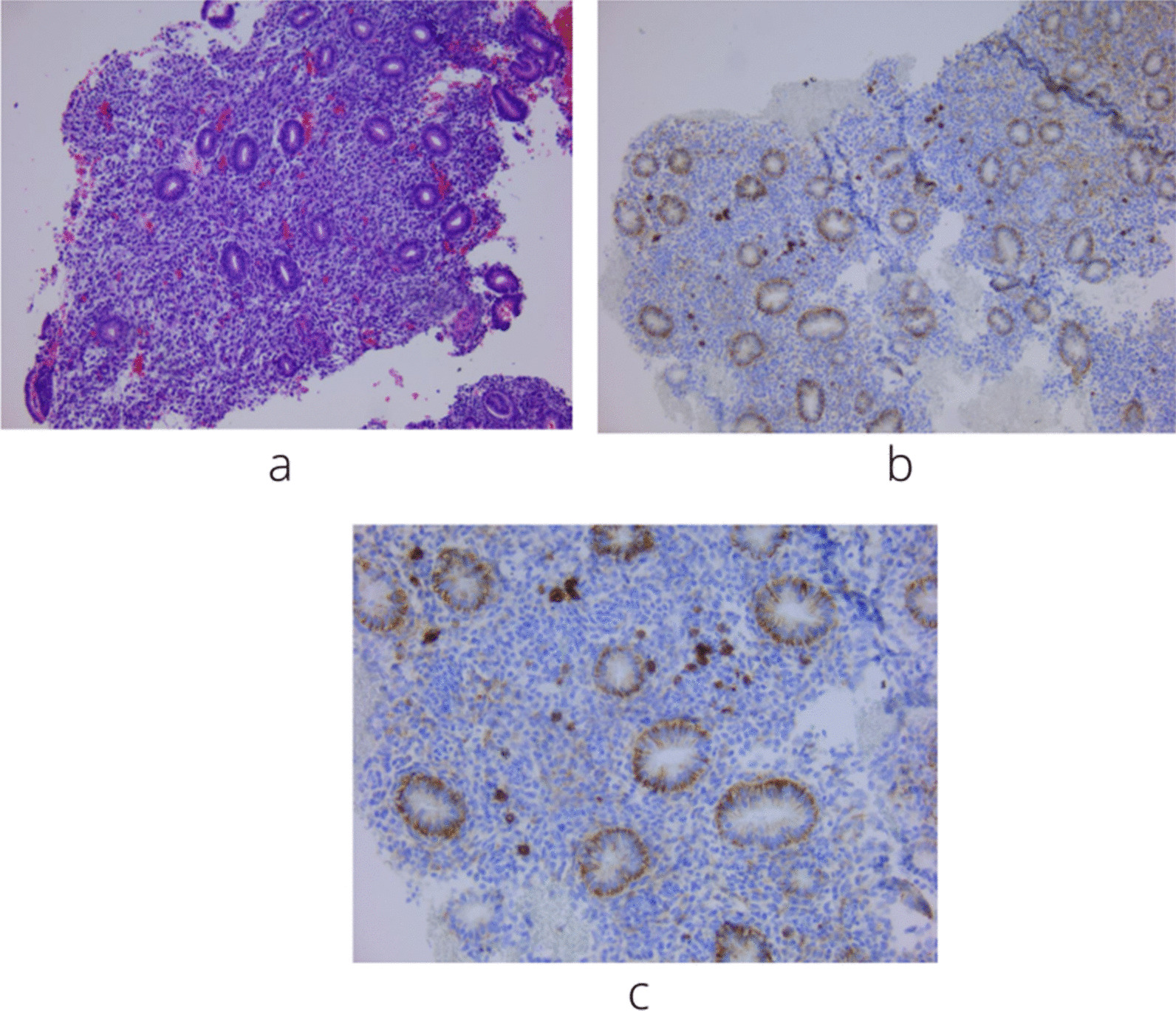
**a**. Fragment of the endometrial mucosa showing proliferative endometrial glands with an edematous stroma. It is difficult to identify plasma cells in HE staining. Most inflammatory cells are lymphocytes (HE 100X). **b**. Fragment of the endometrial mucosa that shows a positive reaction to CD138 by the IHC (immunohistochemical) technique. Positive control is represented by glandular epithelial cells. In the stroma there are cells with a positive expression (100x). **c**. The image shows the expression of the positive control (glands) for the CD138 reaction and in the stroma there are several cells with surface marker expression, with equal epithelial intensity of CD138 (plasma cells). Most stromal cells are negative. 200X

The histological evaluation was done by identification of one or more plasma cells in the endometrial stroma. Presence of CD138 positive plasma cells were confirmed by brown colored membranes and cytoplasm [23, 30]. (Fig. 3).

The normal hysteroscopic aspect had a similar prevalence in patients with the immunohistochemical positive CD 138 as in the group that tested negative at the immunohistochemical CD 138 biopsy for chronic endometritis.

The antibiotic treatment had no significant adverse effect either orally or in uterine infusion and the hysteroscopic procedure had no complication including no bleeding or infections.

## Discussions

Our study provides new and clinical relevant information for physicians about the management of chronic endometritis that remains an emerging topic regarding its diagnosis, treatment and its impact on the result of fertility treatments, as much as, frequently the microbiological results of vaginal and uterine environment are negative [31].

The hysteroscopic examination as a diagnostic tool is used by many practitioners [32, 33], but as our study shows the endometrium hysteroscopic view is a vague diagnosis criteria [34], the normal hysteroscopic aspect did not differ significantly between the positive CD 138 and negative CD 138 groups. This finding proves the importance of endometrial biopsy testing in all patients, even if the hysteroscopic view appears normal.

Although there is no unanimously accepted definition of CE, pathologists agree that using IHC to determine plasma cells by labeling CD138 is currently the most specific and reliable method. Improvement of the diagnosis rate in CE is achieved by implementation of IHC techniques [35]. Besides the true plasmacytes, there are also cells with a plasmacytoid appearance (mononuclear and plasmacytoid stromal cells) which in case of using basic HE stain can be confused with the plasma cells. Additional IHC techniques decrease the false-positive rate, which leads to less overtreatment in women [6]. All published studies show that immunohistochemistry for CD138 positive cells is a golden standard in CE [20]. Although there is no consensus on the IHC assessment of plasma cells, the vast majority of authors believe that the presence of a single cell is sufficient for the diagnosis of CE. Other interpretations used to evaluate CD138 are related to the variable number of plasma cells or the number of HPF images taken into account, from one plasma cell on HPF to 5 plasma cells on 10 HPF or from at least one plasma cell on 10 HPF to 5 plasma cells on 20 HPF [18, 20, 21, 36].

However, the use of HPF-related assessment is restrictive and arbitrary in some ways. In the case of small biopsies, the HPF report may lead to false-negative results. Inclusion of only 10 HPFs may not be sufficient to produce a consistently reproducible result, because in most cases the number of plasma cells present in the endometrial stroma is low. From the histological point of view, the most accepted definition of CE is the presence of a single cell labelled CD138 in the entire surface of the sample [27, 35] and we agree with this hypothesis.

Prevalence in our study was 49.6% but we took into consideration an infertile population requiring an IVF procedure and we used strict diagnostic criteria. Other authors have also published high prevalence values regarding specific groups: from 9 to 56% in recurrent miscarriage [15, 20, 37] and from 14 to 57.3% in patients with recurrent implantation failure [12, 21, 35].

The main of observation criteria is the negative immunohistochemical field after different treatment options: oral antibiotic administration and intrauterine infusion. To our knowledge there are two publications that describe the method of intrauterine antibiotic infusion: a case report [22] that observes the disappearance of clinical and paraclinical findings in chronic endometritis after intrauterine antibiotic infusions in patients that did not respond by oral antibiotics and a randomized trial [38] that compares the oral antibiotic therapy and the simultaneous oral antibiotic therapy with intrauterine antibiotic infusion. Similarly to the previously mentioned studies, our research shows better results in attaining a negative CD138 immunohistochemical finding after the intrauterine antibiotic infusion as a treatment option when compared to oral antibiotic treatment.

### Study limitations

Being a unicentric prospective study with strict inclusion criteria and the necessity of a positive diagnosis of chronic endometritis, the number of cases will be limited, which will lower the statistical power of conclusions. The treatment with antibiotic in uterine infusions is recent in practice and we have considered that the acceptance of patients is mandatory, which excludes the possibility of randomization leading to inequal groups and error sources.

## Conclusion

Based on our findings there is a high prevalence of chronic endometritis in infertile patients and screening for it is useful and should be implemented. In the vast majority of the cases we obtained negative results after antibiotic treatment. This is an argument for the infectious nature of chronic endometritis. Antibiotic treatment administered through intrauterine infusions is much better than oral antibiotic treatment for curing chronic endometritis. Accordingly future studies on chronic endometritis therapy involving untreated control are probably discouraged for ethical reasons.

## Data Availability

The datasets used and/or analysed during the current study available from the corresponding author on reasonable request.
